# Pathological Diagnosis with a Whole Slide Imaging System

**DOI:** 10.1155/2014/690804

**Published:** 2014-12-17

**Authors:** Daiki Taniyama, Kazuya Kuraoka, Akihisa Saito, Miho Tanaka, Yoko Kodama, Junichi Sakane, Yukari Nakagawa, Naoko Yasumura, Toshinao Nishimura, Kiyomi Taniyama

**Affiliations:** ^1^Department of Diagnostic Pathology, National Hospital Organization Kure Medical Center and Chugoku Cancer Center, Kure, Hiroshima 7370023, Japan; ^2^Institute for Clinical Research, National Hospital Organization Kure Medical Center and Chugoku Cancer Center, Kure, Hiroshima 7370023, Japan; ^3^National Hospital Organization Kure Medical Center and Chugoku Cancer Center, Kure, Hiroshima 7370023, Japan

## Background

Whole slide imaging (WSI) is increasingly popular in pathology. At our institute, we utilize a WSI system, an automated slide preparation (ASP) system, an autostainer, and specified software as an ancillary method in making pathological diagnoses of samples submitted as routine work.

## Methods

*Diagnosis*. A VS800 (OLYMPUS, Tokyo, Japan) in a WSI system and an AS-400 (KURABO, Tokyo, Japan) for the ASP system were used. Up to 100 slides consisting of biopsy, endoscopic mucosal resection (EMR), or endoscopic submucosal dissection (ESD) specimens were scanned and stored digitally in one set and the digital images were viewed on a monitor in a separate room using a VS800 viewer. Ocular observation using a conventional microscope was used when necessary to look at the specimens directly. One hundred sixty-six biopsy specimens were analyzed to evaluate the basic ability of the current WSI system in making a pathological diagnosis.

*Autoanalysis*. Immunohistochemistry (IHC) findings for breast cancer samples by a labeled streptavidin biotinylated-peroxidase (LSAB) method using a Benchmark XT (Roche, Basel, Switzerland) were analyzed automatically using a NanoZoomer 2.0-HT (Hamamatsu Photonics, Hamamatsu, Japan) as a WSI system and specified software (Genie, Vista, CA) for image analysis. Twenty-five breast cancer specimens were autoanalyzed to provide IHC of estrogen receptor (ER), progesterone receptor (PgR), HER2, Topoisomerase (Topo) II alpha, and Ki-67. The results were compared with those by ocular observation.

## Results

*Diagnosis*. Of 166 biopsy samples, direct ocular observation was necessary in only 13 (7.8%) to confirm the pathological diagnosis that was made by the WSI system. Difficulty in making a diagnosis was found in seven (4.2%) samples, and inadequate focus or scanning in small parts of specimens was observed in 19 (11.4%) samples. EMR and ESD specimens were also scanned. In addition to HE stain, EVG stain or D2-40 immunostain was also scanned and viewed on the same screen when necessary. Comparison of different stains for the same area became much easier on one or two separate monitors in the WSI system.

*Autoanalysis*. For all antibodies, except for HER2, concordant results were obtained in 24 ER positive cases. The Ki-67 index (*r* = 0.96) and Topo II alpha index (*r* = 0.95) also showed a significant correlation (*P* < 0.001). For HER2, all four specimens with a Hercep-score of two by ocular observation, and one by autoanalysis, revealed no HER2 gene amplification.

## Conclusion

Pathological diagnosis utilizing a WSI system is useful, although there continue to be some issues that need to be addressed. The transference of a whole histological slide into digital form enables the evaluation and interpretation of pathology samples with analytical software in a manner where several observers can join together or by an individual at any time. Well-organized autoanalysis is more likely to result in an objective observation and provide a means by which we standardize IHC methods for breast cancer.

